# The Role of Tumor Necrosis Factor α in the Biology of Uterine Fibroids and the Related Symptoms

**DOI:** 10.3390/ijms19123869

**Published:** 2018-12-04

**Authors:** Michał Ciebiera, Marta Włodarczyk, Magdalena Zgliczyńska, Krzysztof Łukaszuk, Błażej Męczekalski, Christopher Kobierzycki, Tomasz Łoziński, Grzegorz Jakiel

**Affiliations:** 1 Second Department of Obstetrics and Gynecology, The Center of Postgraduate Medical Education, 01-809 Warsaw, Poland; 2 Department of Biochemistry and Clinical Chemistry, Department of Pharmacogenomics, Medical University of Warsaw, 02-097 Warsaw, Poland; mdwlodarczyk@gmail.com; 3 Students’ Scientific Association at the I Department of Obstetrics and Gynecology, Medical University of Warsaw, 02-015 Warsaw, Poland; zgliczynska.magda@gmail.com; 4 Department of Obstetrics and Gynecological Nursing, Faculty of Health Sciences, Medical University of Gdansk, 80-210 Gdansk, Poland; krzysztof.lukaszuk@invicta.pl; 5 INVICTA Fertility and Reproductive Center, 80-172 Gdansk, Poland; 6 Department of Gynecological Endocrinology, Poznan University of Medical Sciences, 60-513 Poznan, Poland; blazejmeczekalski@yahoo.com; 7 Division of Histology and Embryology, Department of Human Morphology and Embryology, Wroclaw Medical University, 50-368 Wroclaw, Poland; ch.kobierzycki@gmail.com; 8 Department of Obstetrics and Gynecology Pro-Familia Hospital, 35-001 Rzeszów, Poland; tomasz.lozinski@pro-familia.pl; 9 First Department of Obstetrics and Gynecology, The Center of Postgraduate Medical Education, 01-004 Warsaw, Poland; grzegorz.jakiel1@o2.pl

**Keywords:** uterine fibroid, leiomyoma, tumor, tumor necrosis factor α, cytokine, growth factor, inflammation, clinical symptoms, pathophysiology, therapy

## Abstract

Uterine fibroids (UFs) are the most common benign tumors of the female genital tract. The incidence of UFs has been estimated at 25–80% depending on selected population. The pathophysiology of UFs remains poorly understood. The transformation of smooth muscle cells of the uterus into abnormal, immortal cells, capable of clonal division, is the main component of all pathways leading to UF tumor formation and tumor necrosis factor α (TNF-α) is believed to be one of the key factors in this field. TNF-α is a cell signaling protein involved in systemic inflammation and is one of the cytokines responsible for the acute phase reaction. This publication presents current data about the role of tumor necrosis factor α in the biology of UFs and the related symptoms. TNF-α is an extremely important cytokine associated with the biology of UFs, UF-related symptoms and complaints. Its concentration has been proven to be elevated in women with clinically symptomatic UFs. The presented data suggest the presence of an “inflammation-like” state in women with UFs where TNF-α is a potent inflammation inducer. The origin of numerous symptoms reported by women with UFs can be traced back to the TNF-α influence. Nevertheless, our knowledge on this subject remains limited and TNF-α dependent pathways in UF pathophysiology should be investigated further.

## 1. Introduction

### 1.1. Uterine Fibroids—An Overview

Uterine fibroids (UFs) are the most common benign tumors of the female genital tract. The incidence of UFs has been estimated at 25–80%, depending on the populations and multiple risk factors [1,2,3,4]. A significant percentage of UF-positive women are symptom-free but UFs cause clinical symptoms of sufficient intensity to impair normal daily functioning in about one-third of the affected subjects [2,4,5,6]. The most common symptoms include excessive bleeding and secondary anemia, pelvic discomfort or pain, bowel and bladder dysfunctions, infertility, and obstetric pathologies [1,2,7,8]. Symptomatic UFs are the leading cause of a decreased quality of patient life [6,9], and the main reason behind various surgeries, chief among them hysterectomy [10,11].

### 1.2. Uterine Fibroids—Growth Factors and Steroid Control

**Figure 1:**
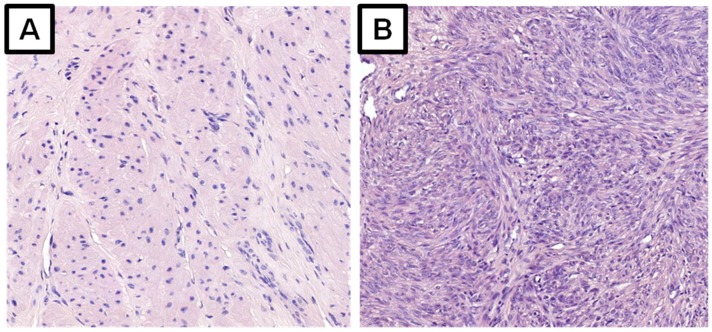
Microscopic slides, histologic specimens of the myometrium (**A**) and a uterine fibroid (**B**). Hematoxylin and eosin stain, 200× magnification.

Despite intensive research, the pathophysiology of UFs remains poorly understood. The transformation of smooth muscle cells of the uterus into abnormal, immortal cells, capable of clonal division, is the main component of all pathways leading to fibroid tumor formation. The second component is tumor growth through uncontrollable cell division, as well as production and accumulation of the extracellular matrix (ECM) [2,7,12,13] (Figure 1).

According to various authors, UF metabolism is affected through steroid hormones, growth factors, cytokines, and chemokines [7,14,15,16,17,18]. Paracrine signaling plays an important role in cellular transformation of the myometrium. The initiators of UF formation are not completely understood, but estrogen and progesterone are believed to be the major promoters of their growth [12,16]. The hormonal effect of these hormones on UFs is related to the aforementioned molecules [7,16]. The influence of steroids on the growth factor expression suggests that these factors represent the ultimate effectors of steroid action [7]. Also, the literature offers some reports suggesting non-genomic interactions between growth factors and hormonal pathways [7,19].

Steroid action is mediated through the interactions of estrogen (ER) and progesterone (PR) receptors with different DNA response elements, which regulate the transcription of selected genes [12,20]. Estrogen and its receptors play an important role in myometrial tissue metabolism and UF growth [12,18,21]. The effect of estrogen on these tumors is evident as these tumors do not appear before the menarche and their size decreases after menopause. It is currently believed that reduced apoptotic potential with increased proliferative potential is associated with the progesterone component rather than with estrogens [12,16,22,23,24]. The main mechanism of action of progesterone in tumorigenesis is its effect on the increase in the concentration of selected growth factors [12,23,24,25]. Disturbances in growth factors (e.g., transforming growth factor β (TGF-β)) [26,27,28,29] and cytokine secretion may be the cause of UF-derived symptoms [7,20,30]. Some of the UF properties might also be regulated by different miRNAs in order to alter their effect on structural homeostasis of female genital tract [31]. Alas, only a small aspect of the complex UF pathogenesis network is known and more evidence is necessary. Extensive worldwide research is ongoing [8].

### 1.3. Uterine Fibroids and the Extracellular Matrix

UF growth is determined by the rate of cell proliferation, differentiation, apoptosis, angiogenesis and ECM deposition [13,20,32]. As mentioned above, UFs are considered to be a type of a fibrotic disorder with excessive ECM production [13,20,33,34]. Fibrosis arises through two pathways: recruitment of the inflammatory cells and activation of the fibroblasts [13,35,36]. Fibroid tumor tissue contains approximately 50% more ECM than the adjacent myometrial tissue. In addition, the architecture of collagen fibrils in UFs has been found to be abnormal [36]. The main fibroid ECM components include collagen (type I and type III), fibronectin, and proteoglycans [13,14,32,34,37]. Normal ECM undergoes a continuous balanced rebuild process, which contributes to the maintenance of its proper amount and density. ECM matrix enzymes are regulated by special tissue inhibitors of metalloproteinases [13,38]. Peptide growth factors can have a regulatory effect only if they bind to their specific receptors and induce signal transmission inside the cell [7,39,40]. This condition is possible only when the factor is released from the complexes with matrix components. ECM accumulation is affected by several factors, e.g., TGF-β, activin A and platelet-derived growth factor (PDGF), TNF-α, followed by estrogen and progesterone [20], and by selected microRNAs [13]. According to Islam et al., ECM can be treated as a reservoir of growth factors and cytokines which protects them from being degraded when staying in the ECM microenvironment [13]. When degraded by matrix metalloproteinases (MMPs), ECM releases soluble forms of various growth factors and cytokines, allowing them to play their molecular roles [13].

### 1.4. Uterine Fibroids and Cytokines

Cytokines are low-molecular-weight proteins which are produced and released by immune system cells [41]. They have a wide range of biological effects and act over short distances, either in an autocrine or paracrine manner [7,42]. Cytokines affect almost all known biological process, including embryonic development, disease pathogenesis, as well as specific and non-specific responses to various antigens and stimuli [41]. They are responsible for intracellular signal transmission by binding to specific surface receptors [41,42,43]. Numerous cytokines have been identified to play a significant role in myometrial and UF biology [35,44,45]. According to Ciarmela et al., interleukins (IL) such as IL-1, IL-6, IL-11, IL-13, IL-15, interferon (IFN)-γ, TNF-α are involved in crucial pathways in the pathophysiology of UFs. The abovementioned cytokines have an effect on the inflammation, neoangiogenesis and the regulation of tissue remodeling [7]. These cytokines may also be responsible for UF-related symptoms, i.e., pain, infertility, and obstetric pathologies [35,46].

### 1.5. Tumor Necrosis Factor α—An Overview and Pathways

Although various molecules are involved in UF biology, it appears that TNF-α may be one of the most important myometrium-associated cytokines [39]. TNF-α is a cell signaling protein involved in systemic inflammation and is one of the cytokines responsible for the acute phase reaction. TNF-α has a dual biological nature as it might cause several undesired effects. TNF-α is a pleiotropic cytokine which has been identified as the key regulator of the inflammatory response [43,47]. It also plays a major role in the cell cycle, being the controller of growth, differentiation, and apoptosis [13,43,47]. TNF-α has multiple biological functions throughout the human body, including fever and acute phase stimulation, promotion of the adhesion molecule expression, phagocytosis stimulation, appetite suppression, and modulation of insulin resistance [43]. TNF-α can be an antineoplastic and antiangiogenic agent which stimulates the immune system to fight cancer cells [39,48]. It has been found that TNF-α is an important gene expression regulator. The interaction of chemokines and their receptors may cause the amplification of cellular signaling pathways and induce the expression of proteins responsible for proliferative cells or changes in their normal metabolism [35,39,49]. Despite the anti-cancer properties of TNF-α, elevated levels of TNF-α are not always capable of destroying all abnormal cells and, paradoxically, they can cause severe symptoms related to tumor occurrence [47,50]. Dysregulation of TNF-α production and distribution has been demonstrated in various human diseases, including cancers, dermatoses, and inflammatory bowel diseases (Table 1) [35,43,51,52].

TNF-α is produced mainly by activated macrophages but it can also be produced by other cell types, e.g., lymphocytes and neutrophils [43,60]. TNF-α is produced as a 233-amino acid-long type II transmembrane protein arranged in homotrimers [61]. The soluble homotrimeric form is then released via proteolysis by the ADAM metallopeptidase domain 17 (ADAM17) and has a triangular shape [62,63]. Importantly, both the secreted and the membrane forms are biologically active, although their specific functions remain the subject of some controversy among the researchers [60,64].

**Figure 2:**
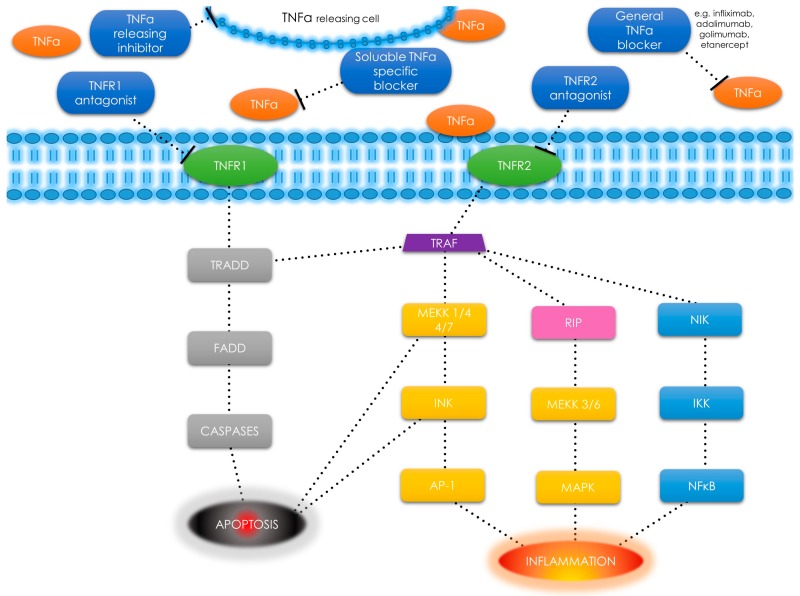
TNF-α receptors, pathways and different types of signals—schematic diagram. TNF-α has an ability to induce apoptosis, cell survival or inflammation depending on selected pathway. Tumor necrosis factor α (TNF-α); tumor necrosis factor α receptor (TNFR); tumor necrosis factor receptor type 1-associated death domain (TRADD); Fas-associated protein with death domain (FADD); tumor necrosis factor receptor-associated factor (TRAF); mitogen-activated protein kinase kinase kinase (MEKK); c-jun N-terminal kinase (JNK); activator protein 1 (AP-1); receptor interacting protein (RIP); mitogen-activated protein kinases (MAPK); nuclear factor κ-light-chain-enhancer of activated B cells (NF-κB); NF-κ-B-inducing kinase (NIK); I κB kinase (IKK).; T-bar as a drug - binding point interaction.

TNF-α is also found in smooth muscle cells as a response to tissue injury or upon immune responses to various stimuli [65,66,67]. TNF-α uses two types of receptors: TNF-α receptor type 1 (TNFR1) and TNFR2 (Figure 2).

TNFR1 is expressed in most tissues, whereas TNFR2 is found primarily in the immune system cells [43]. After binding to the receptor, the TNF-α molecule may activate one of the three potential effects: 1) activation of the nuclear factor kappa-light-chain-enhancer of activated B cells (NF-κB), which is a transcription factor involved in cell survival, proliferation and the inflammatory response (this include NF-κB-inducing kinase (NIK) [68] and I kappa B kinase (IKK) [69]); 2) activation of the mitogen-activated protein kinases (MAPK) pathways (through c-jun N-terminal kinase (JNK) [70] and on the other hand through receptor interacting protein (RIP) kinases family [71] and MAP kinase kinase (MEKK) [72]), involved in cell differentiation and proliferation, and 3) induction of death signaling (Figure 2) [43,60,73,74]. Most of the mentioned pathways are tumor necrosis factor receptor-associated factor (TRAF)2 dependent as presented in Figure 2 [75].

TNFRs form trimers when reached by the ligand. This binding leads to the dissociation of the silencer of death domains (SODD) inhibitory protein [76]. When SODD is finally dissociated, tumor necrosis factor receptor type 1-associated death domain (TRADD) protein binds into free death domain [66,73,77,78]. The TRADD protein binding may activate three different important pathways [66,73,78]. The first pathway is the activation of the NF-κB protein complex, which leads to changes in the DNA transcription, cytokine production and cell survival (Figure 2). The NF-κB pathway is also responsible for cell proliferation, inflammatory response, and anti-apoptotic factors [79]. The NF-κB pathway has some serious connections with the UF-related pathways: the focal adhesion kinase (FAK) signaling activated by TGF-β [29], and with activin A [80]. IL-1α and TNF-α are the factors that block the differentiation of human myoblasts with the activation of the TGF-β-activated kinase (TAK)-1 pathway. As described by Trendelenburg et al., this pathway can be modulated (tracing to p38 and NF-κB) with the use of different factors like drugs or genetic modifications [80]. Additionally, TGF-β increases the expression of apoptosis-related p53 and Bax proteins mediated by their major regulator—TNF-α [81]. Apoptosis is a mechanism by which cells undergo programmed death. This process can be mediated through the receptor or the mitochondrial pathways. The extrinsic pathway involves binding of a ligand e.g., soluble protein like TNF-α to its cognate cell surface receptor [20,82]. The second pathway depends on MAPK activation (Figure 2). TNF-α activates JNKs, which are responsive to stress stimuli. JNKs are involved in various processes, e.g., apoptosis, cell degeneration, differentiation and proliferation, inflammatory conditions, and cytokine production [83,84]. JNKs and p38 MAPKs can exert antagonistic effects on cell proliferation and survival. This crosstalk is an important regulatory mechanism in numerous cellular responses [85]. The third and the weakest pathway is responsible for induction of death signaling (Figure 2). In this pathway, TRADD binds FADD, which is a stimulus to caspase-8 concentration raise and results in subsequent autoproteolytic activation and effector caspases cleaving [86]. These three TNF-α pathways have many conflicting effects. Some of them enhance the transcription of the anti-apoptotic proteins, while others have a positive effect on the inhibitory proteins which interfere with death signaling. This delicate balance can shift both ways, depending on cell type, concurrent stimulation of other cytokines, and the influence of reactive oxygen species [43,73].

## 2. Material and Methods

A review of the publications on the role of TNF-α in UF biology and UF-derived symptoms is presented. Authors conducted their search in PubMed of the National Library of Medicine and Google Scholar. Databases were extensively searched for all original and review articles/book chapters using keywords (one or in combinations): uterine fibroid; uterine leiomyoma; tumor necrosis factor α published in English until October 2018. Moreover additional articles in bibliographies of reviewed articles were searched. Overall, most relevant articles were reviewed and included as appropriate.

## 3. Discussion

### 3.1. Uterine Fibroids and Inflammation

Inflammation plays a major role in tumorigenesis and inflammation-related factors are key players in the development of many benign and malignant neoplasms, especially due to high increase in the occurrence of a mutation and the proliferation rate of the mutated cells [35,87]. As stated by Wegienka, local chronic inflammation constitutes a microenvironment which enables UF development and UFs are the effect of the presence of various inflammatory-derived molecules in the myometrium [88,89]. UF is thought to be an inflammatory-related fibrotic disorder. According to the available data, UF pathophysiological pathways depend on the activated macrophages, whose number in the UF tissue is increased as compared to the adjacent normal myometrium [90]. The abovementioned cells play a key role in the reparative processes and myofibroblast recruitment [35,91]. Menstruation, infections, mechanical injuries, and oxidative stress may be the causes of the inflammation in the uterus [13]. Myofibroblasts produce ECM as a response to the influence of various cytokines and growth factors. In normal conditions, this process can repair damaged tissues. However, it may also lead to fibrosis if there is no control or the deregulation of the controlling pathways is too high [13,32,92,93,94].

**Figure 3:**
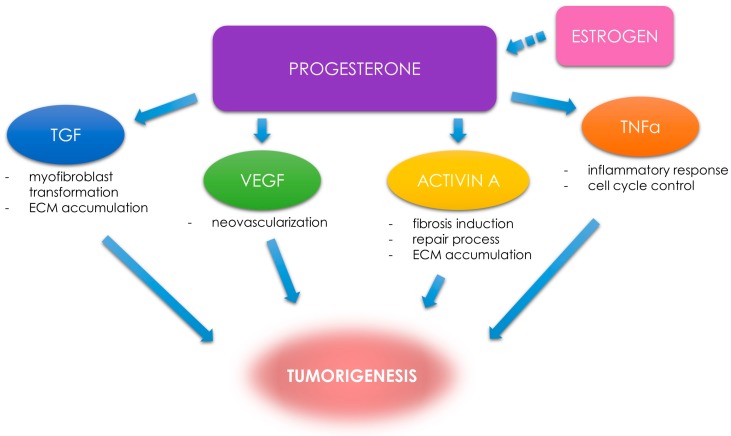
Progesterone and progesterone-related factors. The role of selected factors in UF tumorigenesis. Transforming growth factor (TGF); vascular endothelial growth factor (VEGF); tumor necrosis factor α (TNF-α); extracellular matrix (ECM). Estrogen as a factor preparing the tumor to be stimulated by progesterone (dotted arrow).

MED12 somatic mutation is the most often detected DNA mutation in human UFs (70–80%) [95,96,97,98]. This mutation is a driver for stimulating the development of UFs and influencing genomic instability [99,100]. MED12 is also related to endocrine and growth factor pathways [12]. A possible impact of altered signaling in UF pathophysiology on inflammatory responses and genomic repair mechanisms is being extensively researched [101]. UFs growth is mostly hormone-dependent and progesterone is believed to be the most important hormone in this complicated process [12,23,24]. The main mechanism of progesterone action in UF tumorigenesis is its effect on the increase in the concentration of selected growth factors [7,29]. Factors which have been proven to have a serious impact on UF tumorigenesis include TGFs, activin A, vascular endothelial growth factors (VEGFs), and other pro-inflammatory agents [14,15,29,102] (Figure 3).

According to the available sources, elevated levels of TGF-β play an important role in UF growth and clinical symptom progression [7,26,27,29,103], which appears to be one of the key factors in myofibroblast transformation and fibrosis progression [17,29,35,37]. Activin A is yet another factor, by no means less important, which was found to be involved in cell proliferation, differentiation, death signaling, and metabolism [81,104]. Activin A and the related proteins, e.g., anti-Müllerian hormone (AMH) or bone morphogenetic proteins (BMPs), belong to the TGF-β superfamily [105]. Recent studies have proven the role of activin A in inflammation processes [35,81,105], wound repair [106], and fibrosis [81,107]. Activin A produced by macrophages is responsible for cell transformation, which leads to tumor occurrence [35,108]. It also plays various physiological roles in a wide range of other tissues [109]. As suggested by different authors, elevated activin A concentrations might also be responsible for excessive ECM production [81,104]. In a study by Ciarmela et al., mRNA levels of activin A were found to be more expressed in UF tissue as compared to the adjacent myometrium [109]. Islam et al., found that mRNA levels of collagen, fibronectin and versican variants in ECM were increased by activin A [104]. The same authors also found that activin A induced phosphorylation of the Smad proteins in UF cells [104], which is one of the pathways of profibrotic signaling [13,29]. The potential pathological connections between TGF-β, activin A and TNF-α will be described later in this article.

### 3.2. ECM and Inflammation in Uterine Fibroids

ECM found in UF differs from the normal ECM in a well-formed myometrial tissue [13,14,110]. The presence of inflammatory cells in UFs contributes to excessive ECM production, tissue remodeling, and tumor growth [35]. ECM accumulation is regulated by growth factors, cytokines and steroid hormones. Increased production and accumulation of abnormal ECM results in further tumor volume gain [13,14,110]. MMPs are the enzymes which are responsible for matrix lysis and remodeling [29,111,112,113]. They are regulated by tissue inhibitors of metalloproteinases (TIMPs) [38]. Some growth factors increase the concentration of TIMPs, which slows down the conversion of the entire ECM and results in its excessive accumulation [29,103,114]. Modified MMP activity is then insufficient to degrade the appropriate amount of ECM to maintain tissue [29,111,115]. Proteolytic enzymes which are produced as a response to inflammation are responsible for several major UF developing processes, e.g., angiogenesis and proliferation of fibroblasts [20,35]. TGF-β, activin-A, and TNF-α are able to increase the synthesis of ECM components through the activation of multiple signaling pathways, e.g., the Smad proteins and various kinases. Cytokines (e.g., interleukins and TNF-α) and growth factors (e.g., VEGF), fibroblast growth factors (FGFs), endothelial growth factor (EGF), and different TGFs are among those angiogenic factors which have been extensively described due to their ability to regulate the expression of proteases and their inhibitors, and enhance cell proliferation and migration [20,112,115,116]. As stated by Chegini (2010), angiogenesis depends on the specific balance between promoters and inhibitors [20]. In this place, TNF-α plays the role of the angiogenic suppressor [20], but its role is much more complex.

### 3.3. Tumor Necrosis Factor α in Uterine Fibroids

TNF-α is produced by macrophages whose significant numbers can be found in UFs [35,43,60]. Increased TNF-α expression has been found in UF tumors as compared to the adjacent normal myometrium [49]. According to Nair et al., TNF-α secreted by adipocytes enhances the proliferation of UFs [117]. What is currently known is that polymorphisms in the genes encoding IL-1β, IL-6, and TNF-α have been associated with an increased risk of these tumors [20,118,119,120].

According to available data, fibroid tissue and the adjacent unchanged myometrial tissue demonstrated that TNF-α is abundantly present in the cytoplasm of tumor cells [49,121], which is consistent with the findings of Plewka et al., from 2013 [122]. In that study, Plewka et al., investigated the expression of different inflammatory mediators, e.g., IL-1β, TNF-α or cyclooxygenase 2 (COX-2), in the normal myometrium and UFs in women of reproductive age. These authors found significantly higher TNF-α immunoreactivity in UFs as compared to normal uterine smooth muscle tissue [122].

In the work of Kurachi et al., staining for TNF-α in UF tissue obtained in the proliferative phase was more abundant than in the secretory phase of the menstrual cycle [49]. This relation was not found in the normal myometrium [49]. The addition of progesterone resulted in a decrease in immunoreactive TNF-α expression as compared to control cultures, while a similar addition of estradiol did not affect the TNF-α expression [49]. In cultured UF cells, the treatment with progesterone inhibited the expression of insulin-like growth factor 1 (IGF-1) and the TNF-α, and augmented the expression of apoptosis-inhibiting Bcl-2 protein [123], which is consistent with other reports in the literature. Progesterone is a steroid hormone with anti-inflammatory and mitogenic activity [101,124]. Natural killer (NK) cell activity is suppressed under the influence of progesterone [125]. Progesterone regulates uterine NK cells through a glucocorticoid receptor mediated process and steroidal antiprogestogens (e.g., mifepristone) could abolish the inhibitory effect of this hormone [126]. Elevated levels of progesterone have an effect on increased TGF-β and decreased TNF-α production [7,29,101]. Progesterone, being the main steroid hormone responsible for the formation and growth of UFs, should lead to a decrease in TNF-α concentration. However, paradoxically, the remaining pathways, many of which are still undefined, make TNF-α concentrations higher in the presence of these tumors, as shown by other authors [39] and our recent work [30].

The picture becomes more complex when we bear in mind that TNF-α is a potent stimulator of aromatase activity, what results in enhanced conversion of androstenedione to estrone [127]. TNF-α influences key genes and enzymes involved in estrogen metabolism, making it more hormonally active and carcinogenic [128]. Estrogens have been known to play a major role in UF pathophysiology as described above, including also pathways like Ras-Raf-MEK, which are more in common with TNF-α [18].

The reciprocal feedback of progesterone and TNF-α seems more complex when considered along with activin A. As mentioned before, activin A is one of the major factors involved in UF pathogenesis, with a direct pro-fibrotic effect on UF cells by the expression of ECM protein (via Smad pathway) induction [104]. The regulation of activin A biological function in fibrosis-related processes is complex due to the influence of several molecules [81]. The effect of TNF-α in the paths in which activin A is involved is vital, but the pathophysiology of this factor involves also TGF-β1, IL-1, IL-1β, IL-13, angiotensin, and others [81,129,130]. In a study by Protic et al., activin A mRNA expression was upregulated by TNF-α in myometrial and UF cells [35]. The same effect was also found in other cell types [129].

Activins are produced in the gonads, pituitary gland, placenta, and other organs. In the ovary, activin increases FSH binding and FSH-induced aromatization. Activin A enhances the activity of aromatase enzyme and simultaneously suppresses progesterone production [131]. In a study by Shukovski and Findlay (1990), activin A was found to delay the process of luteinization [132]. Hillier et al., concluded that activin A is responsible for promoting estrogen synthesis and simultaneously suppresses the synthesis of progesterone [131]. According to Ciarmela et al., activin A should be considered as a steroid-regulated factor involved in myometrial functionality and that the disruption of its signaling may contribute to tumor growth [109]. The abovementioned observations both, the complexity of these processes and the limitations of the available data. The presented information suggests that UFs have their own complicated path of connections and conjugations, in which TNF-α plays a role as an activin A upregulator, where activin A affects progesterone, which in turn inhibits TNF-α. It seems reasonable to assume that includes one or more additional factors which allow for TNF-α to bypass the inhibitory effect of progesterone and induce its related inflammatory reactions. These observations are also supported by some of the effects of ulipristal acetate (UPA) on UF tumors [133]. Despite the fact that UPA has a potent effect on one of the major UF pathophysiological pathways—progesterone and is TGF-β dependent [27], it also increases the activity of alkaline phosphatase, upregulates caspases, and downregulates TNF-α expression [134]. According to Ciarmela et al., UPA was also found to inhibit the expression and functions of activin A and activin receptor in UF tumor cells [135]. In our opinion, more research about the described pathway and its molecular connections is necessary to better understand it in the context of UFs.

Wang et al., found that TNF-α upregulates the mRNA levels of MMP-2 in UF cell cultures. The same observations were made with protein levels, whereas this effect was insignificant in the normal myometrium [136]. Their finding might play a major role in releasing soluble forms of various growth factors and cytokines from ECM, as mentioned before [13]. Such dependence may also have an effect in the form of a self-winding dependency circle—TNF-α, which releases various cytokines also releases further TNF-α molecules and, at some point, this can become a cascade difficult to control.

Islam et al., found that activin A expression was increased under the influence of TNF-α [16], which might solve the mystery how this cytokine can stimulate ECM production, as activin A is an important profibrotic factor in UF pathology [13,81]. The role of TNF-α in UF formation and growth seems even more justified as it was proven that TNF-α has a potent influence on extracellular signal–regulated kinases (ERKs) [13,136]. The abovementioned ERK pathway plays an important role in integrating external signals from the presence of mitogens and is a part of the Ras-Raf-MEK-ERK signal transduction cascade. This cascade participates in various processes such as cell cycle progression, cell migration, survival, differentiation, proliferation, and transcription [137]. The activity of the Ras-Raf-MEK-ERK cascade is increased in more than one-third of human neoplasms, and inhibition of its components might be an effective anti-tumor strategy [137].

### 3.4. Obesity, Inflammation and Tumor Necrosis Factor α in Uterine Fibroids

Obesity is a condition in which excessive body fat may interfere with the maintenance of an optimal state of health. Obesity is predominantly caused by excessive food intake, lack of physical activity, and genetic susceptibility [138,139]. According to the available data, obesity is considered to be a major risk factor for UFs [3,8,26,140,141]. The reasons for this dependence are in this case sought in the metabolic function of adipose tissue [141,142]. Most adrenal androgens are metabolized to estrogen with aromatase in the adipose tissue [142,143]. Pathophysiological factors attributed to the occurrence of UF include reduced production of sex hormone-binding globulin (SHBG) in obese women [141,144]. This protein binds a large proportion of the circulating sex hormones, not allowing their hormonal activity on sensitive tissues, which affects the delicate hormonal balance of the body [144,145].

Obesity is characterized by a chronic state of inflammation and this might be a cause of abnormal tissue regeneration, as is the case in UFs [35]. Excessive adipose tissue may release various inflammatory mediators. These relations may predispose to a pro-inflammatory state of the tissues and enhanced oxidative stress [146]. Excessive fat accumulation is associated also with increased levels of reactive oxygen species (ROS), which inhibit cell apoptosis and increase ECM deposition [147,148].

Macrophages are important components of the adipose tissue, which actively participate in its activities [35,60,149]. Other immune system cells, like lymphocytes taking part in the metabolism of the adipose tissue, can also lead to immune deregulation [149]. As described in the available studies, adipose tissue produces and releases a variety of pro- and anti-inflammatory factors, including adipokines, as well as various cytokines and chemokines, such as different interleukins and chemoattractant proteins [35,146,150]. Human adipose tissue secretes TNF-α, which may be a good explanation of the relation between obesity and inflammation [151]. In a study by Hotamisligil et al., TNF-α was found to be highly expressed in the adipose tissue of obese people [152], whereas Zaragosi et al., described the same dependence for activin A [35,153]. These inflammation enhancing factors which are accumulated and released by adipose tissue and have a direct effect on the myometrium and myofibroblasts may result in excessive production of the ECM components, tissue remodeling, and UF occurrence [35,81].

Further studies are necessary to show the effect of body weight change (gain or loss) on TNF-α and other pro-inflammatory factors levels, and whether this can affect the symptoms associated with UFs.

### 3.5. Tumor Necrosis Factor α, Uterine Fibroids and the Related Symptoms—Overview

TNF-α may be considered as a chemokine which could influence numerous clinical symptoms associated with UFs [20,35]. Abdominal and pelvic pain, infertility or gastrointestinal complaints are just a few ailments which may be caused not only by tumor pressure but also by the paracrine and endocrine influence of the tumor. In this section some links between UFs-derived complaints and TNF-α are described.

#### 3.5.1. Pain

Symptomatic UFs can cause chronic pelvic pain. Interestingly, this pain cannot always be attributed to the presence of the mass or position of the tumor. While the subject of chronic pain in patients with UFs has been examined only to a small extent, in the case of endometriosis this topic is relatively well-researched [154]. TNF-α levels in the peritoneal fluid are higher in women with endometriosis (higher stages of endometriosis correspond to higher TNF-α levels) [155,156]. The role of TNF-a as a pain inducer has been well-documented [157,158]. TNF-α stimulates the production of prostaglandins (PG) E2 and F2α [154,159]. PGE2, for example, may play a role in the resolution of the inflammation. Some of its effects on the human body include vomiting, fever, diarrhea, and excessive uterine contraction [160]. Pharmacological therapies, including painkillers or hormones for pain management are used to relieve these symptoms [20]. The literature offers some data about a successful use of anti-TNF-α drug—etanercept—in the treatment of endometriotic implants but, to the best of our knowledge, there is no evidence about the use of this drug in UFs treatment [161]. In our opinion, more research about the potential role of TNF-α on UF-derived pain is needed.

#### 3.5.2. Infertility

The connection between UFs and infertility, together with the potential impact of TNF-α in this regard, should be highlighted.

Infertility is defined as the inability to become pregnant or carry a pregnancy to full term. There are many causes of infertility and many different methods of treatment [162]. The immune mechanisms are among the possible causes of some forms of infertility. Cytokines, like TNF-α, selected ILs and others which trigger a Th1 type immune response, are suspected to play a major role in infertility [163]. According to Wang et al., women with a history of infertility have significantly increased serum TNF-α concentrations in comparison to fertile controls, and TNF-α can potentially serve as an infertility marker [164]. According to Falconer et al., inflammation affects the ovaries and, consequently, the oocyte quality, with less favorable outcomes of in vitro fertilization [165]. TNF-α stimulates the apoptosis of human trophoblast cells and inhibits the proliferation of human trophoblast cells in vitro [166,167]. According to some authors, TNF-α interferes with development of the placenta and trophoblast invasion of the spiral arteries [166,168]. Abnormal production of TNF-α and cytokines which are similar in function has been suggested to cause fetal growth restriction [169]. In a very interesting study by Azizieh and Raghubaty, elevated TNF-α serum levels were correlated with severe pregnancy complications, e.g., recurrent miscarriages, premature rupture of membranes, preeclampsia, and intrauterine growth restriction. These authors concluded that TNF-α must be an important factor in the pathogenesis of these complications [168]. According to Austrian experts, TNF-α concentration, distribution and stimulation period determine whether TNF-α would have a beneficial or adverse effect on the female reproductive system [166]. TNF-α was also found to differ between women with and without the polycystic ovary syndrome (PCOS). According to a meta-analysis by Gao et al., TNF-α serum levels in women with PCOS are elevated as compared to PCOS-free controls. Those authors concluded that TNF-α serum levels might be related to insulin resistance and androgen excess [170].

It is officially accepted that UFs decrease the fertility potential [171,172]. According to Pritts et al., UF-positive women have decreased rates of implantations and live births, and increased rates of spontaneous miscarriages [171]. Some cytokines are thought to be responsible for implantation success and optimal embryonic development [173]. TNF-α may just be one of these cytokines and there is a chance that too high concentrations of TNF-α can have a significant impact on live birth occurrence. In our opinion, reducing the TNF-α induced inflammation in the uterus, e.g., by removing the UFs, might result in successes of the reproductive medicine. Unfortunately, data research on this topic remain limited and further research is necessary. Our study, which demonstrated elevated serum TNF-α levels in UF-positive women, was a step in that direction [30]. The next stage would be to investigate the impact of surgical or drug therapies (e.g., UPA or alternative agents) on the concentrations of selected cytokines and growth factors and find how to achieve the best effect, i.e., successful conception [172,173,174]. Studies about UPA and infertility are ongoing and the first answers are expected soon [175].

#### 3.5.3. Gastrointestinal Issues

TNF-α plays a major role in gastroenterology. Not all gastrointestinal symptoms associated with UFs are only related to pressure on the neighboring organs. Some of them may also have a paracrine or endocrine background. There is increasing evidence for the involvement of the immune system and the related cytokines in various gastrointestinal disorders, altered cytokine expression, and abnormal presence of immune cells, resulting in the occurrence of the clinical symptoms. Several diseases and symptoms depend on the TNF-α-related pathways [176]. TNF-α is overexpressed in patients with colitis ulcerosa and Crohn’s disease, and the degree of mucosal inflammation is positively correlated with chemokine secretion patterns [177]. TNF-α is also a known factor in the initiation and amplification of the inflammatory responses to Helicobacter pylori infection [178,179,180]. TNF-α is a potent inhibitor of gastric acid secretion [179], and its improper secretion might be a causable factor of many cases of dyspeptic disorders in women with UFs.

Therefore, TNF-α targets were proposed as potential treatment options and today anti-TNF-α drugs are the gold standard in inflammatory bowel disease therapy [181,182]. These diseases are treated with the use of TNF-α inhibitors, e.g., monoclonal antibodies like infliximab, adalimumab, and certolizumab [183]. There is also the abovementioned decoy circulating receptor drug, etanercept, which binds to TNF-α [184]. There are some data indicating prolonged disease stabilization in patients treated with the use of TNF-α inhibitors, but most studies found no difference in the efficacy of that treatment when compared to placebo [185]. However, there is a difference between benign UFs and malignant tumors, and further laboratory tests should be performed to evaluate the effects of these drugs on UFs.

### 3.6. Tumor Necrosis Factor α, Uterine Fibroids and the Related Symptoms—Management

UFs are just one serious health problem worldwide. Pharmacotherapy is an important chapter in UF management. Prophylaxis of UFs is practically non-existent, while treatment is often costly and expensive. According to Soave et al. current anti-UF strategies that preserve uterus and fertility are not capable yet of controlling clinical symptoms and tumor progression and they are mostly ineffective in the long term outcome [6]. Anti-UF agents should be chosen according to the size and location of UFs, age, dominant symptoms, childbearing plans and treatment availability [186]. Potent drugs like gonadotropin-releasing hormone (GnRH) analogs or selective progesterone receptor modulators (SPRMs) are an option for patients who need symptom relief preoperatively, who are approaching menopause or do not accept surgical intervention. GnRH analogs have been used in women with UFs to reduce bleeding and tumor volume, but their use is limited to short term due to their hypoestrogenic side effects [186]. The other drugs recently used in UF therapy are SPRMs, drugs which interact with progesterone receptors. SPRMs [187] both treat symptoms and eliminate or delay the need of surgery [6,188].

**Figure 4:**
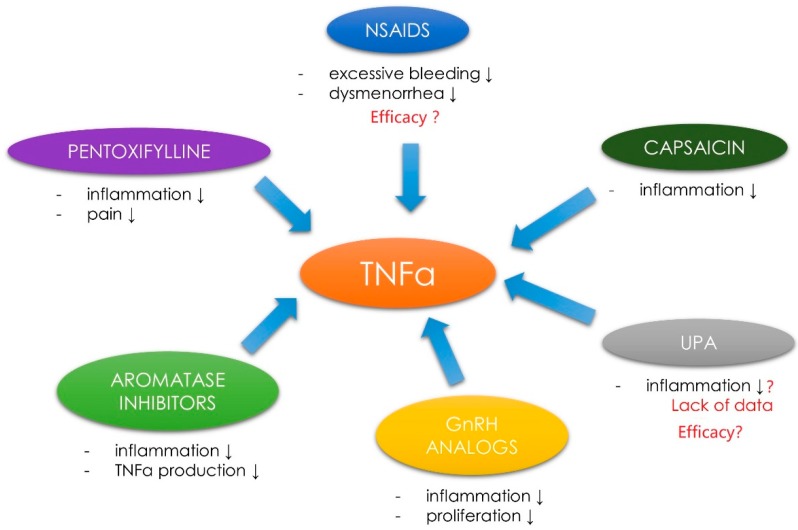
Drugs with proven and potential effect against TNF-α in UF therapy. Nonsteroidal anti-inflammatory drugs (NSAIDS); tumor necrosis factor α (TNF-α); ulipristal acetate (UPA); gonadotropin-releasing hormone (GnRH); ↓ as decrease.

In the case of other anti-UF agents, their poor effectiveness remains the greatest issue. Innovative forms of UF pharmacotherapy are still under intensive investigation. As stated in recent review from 2017 vitamin D, paricalcitol, epigallocatechin gallate, elagolix, aromatase inhibitors (AIs) and cabergoline might find their place in UF therapy as safe and effective alternatives or co-drugs [174]. After reviewing the current literature authors found that anti-TNF-α may be also a promising option in the nearest future. Further research on this topic should focus on individually tailored strategies, where for example anti-TNF-α drugs would be used in women with UF-dependent clinical symptoms that occur due to TNF-α pathways dysregulation. In light of the above, it would seem prudent to assume that drugs directed against TNF-α would be applicable for the relief of UF-derived symptoms (Figure 4).

However, this is not so obvious due to the failed attempts to use anti-TNF-α drugs (like infliximab) in case of endometriosis-related pain due to the lack of clinical effects [189,190]. Better results were obtained with pentoxyfilline, a methylated xanthine derivative, a competitive nonselective phosphodiesterase inhibitor [191]. Pentoxifylline inhibits TNF-α production in vitro and reduces the inflammatory action of this molecule [192,193]. According to a study by Kamencic and Thiel, postoperative treatment with pentoxifylline reduced the pain in patients with endometriosis [193]. To the best of our knowledge, there are no studies about the use of pentoxyfilline in UF therapy. However, this might be an interesting idea for future studies on UFs (Figure 4).

Nonsteroidal anti-inflammatory drugs (NSAIDs) are widely used to treat endometriosis-TNF-α-related symptoms. Nevertheless, data proving that they significantly reduce endometriosis-related pain remain insufficient [194,195]. According to some sources, NSAIDs can be used to reduce excessive bleeding and dysmenorrhea symptoms, but there are not enough data about their efficacy [196] (Figure 4).

According to less known studies, capsaicin and its analogs are able to block TNF-α-induced NF-κB activation in a dose-dependent manner. This substance can therefore play a chemopreventive role in tumor growth and the derived symptoms. However, no studies on that topic are available [197] (Figure 4).

The use of AIs in therapy has given rise to some expectations [174]. This therapy also includes some TNF-α-dependent pathways. AIs decrease the production of estrogens by blocking or inactivating aromatase [174]. UF tissue expresses aromatase in higher amounts than normal myometrial tissue [143,198]. Some connections between aromatase and TNF-α have been confirmed. Cytokines, like ILs, prostaglandins and TNF-α can stimulate aromatase activity [127,199]. For example, TNF-α induces aromatase expression via the adipose specific promoters [200]. To et al., found that TNF-α regulates estrogen biosynthesis within the breast, affecting the activity of the key estrogen-derived enzymes, i.e., aromatase or estrone sulfatase. These authors stated that TNF-α targeting might be useful as a novel approach to anti-tumor therapies [201]. As described in our recent review on alternative agents in UF therapy, AIs are potent drugs in UF management which reduce UF volume and improve the associated symptoms, probably also by the reduction of TNF-α production (Figure 4). Unfortunately, data about AIs use in UF treatment are incomplete and more studies are still necessary [174]. Last but not least, GnRH analogs are drugs used in UFs management which affect the TNF-α pathways. GnRH analogs act by binding to GnRH receptors, which leads to hypogonadotropic hypogonadal state [202]. GnRH agonists have a direct and indirect effect on the tissue in reducing proliferation and improving clinical symptoms [202]. Some of their effects might be explained by TNF-α-related pathways. In a study by Taniguchi et al., GnRH analog treatment attenuated TNF-α-induced cell proliferation in the endometrial stromal cells [203]. More evidence has come also from other Japanese authors who showed that GnRH analogs treatment attenuated the expression of IL-8 by reducing TNF-α-induced NF-κB activation [204]. In an interesting study about spinal cord inflammatory response, Guzman-Soto et al., found that GnRH analog—leurolide has an effect on the activation/expression levels transcription nuclear factor NF-κB and the proinflammatory cytokines like TNF-α [205]. Further research about the connections between GnRH analogs and TNF-α pathways is needed (Figure 4).

UPA, one of the main drugs related to the treatment of UFs [6,133,188] cannot be omitted. Data on UPA and its effect on inflammation are incomplete. We know many paths of action of UPA, both biochemical and genetic [133,206,207]. In the studies published by our team, we obtained preliminary results that UPA may influence some growth factors, e.g., TGF-β3 [27]. Research on other factors is ongoing and the results will be presented in the nearest future. In their study on the effect of UPA on the human endometrium, Whitaker et al., found that there were significant changes in insulin growth factor binding protein 1, IL-15, HOXA10 mRNAs expression as compared to controls. What is more, cell proliferation in UFs from the UPA group was lower than in women in the proliferative phase [208]. It might suggest some connection between UPA and inflammation. UPA has a potent effect on progesterone-TGF-β pathway [27], it also increases the activity of alkaline phosphatase, upregulates caspases and downregulates TNF-α expression [134]. There are no studies on the connections between TNF-α and UPA (Figure 4).

### 3.7. Tumor Necrosis Factor α—Novel Concepts in Diagnosis and Therapy

In our opinion, there is a possibility that TNF-α serum levels may become a marker used for clinical verification in the case of problematic differentiation (UF or different tumor) [30], or to determine the risk of clinical symptoms, as can be partially done with endometriosis [209].

**Figure 5:**
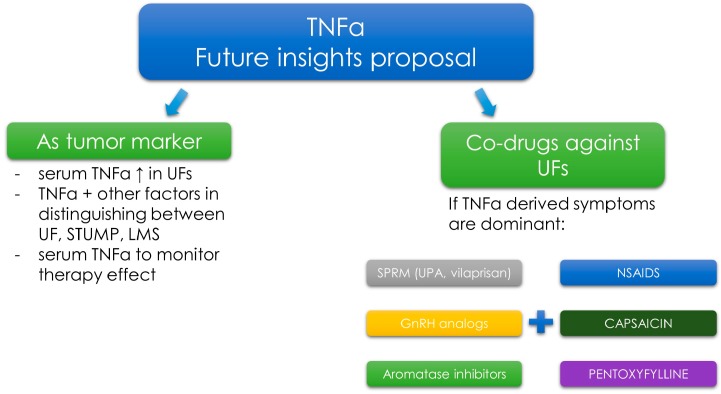
Potential future directions in UF diagnosis and therapy with the use of TNF-α. Tumor necrosis factor α (TNF-α); uterine fibroid (UF); smooth muscle tumor of uncertain malignant potential (STUMP); leiomyosarcoma (LMS); selective progesterone receptor modulator (SPRM); ulipristal acetate (UPA); nonsteroidal anti-inflammatory drugs (NSAIDS); ↑ as increase.

According to a study by our group, women can be diagnosed with UFs solely using the serum TNF-α level cut-off point [30]. Studies on TNF-α levels in patients with diseases similar to UFs, such as uterine sarcoma or adenomyosis, may open up a new chapter in gynecological diagnostics (Figure 5).

If higher levels of TNF-α are confirmed in the serum of patients with uterine sarcoma, as compared to UF-positive patients, TNF-α could be considered as a non-specific marker which could indicate factors such as what type of surgery should be chosen, if morcellation can be performed, and where to refer the patient. This is important, especially in the current state of surgical management of UFs [210]. TNF-α might be useful marker to estimate the risk of UF occurrence, or for the evaluation of treatment effectiveness (e.g., in UPA therapy). Due to the complexity of the pathophysiological pathways in which it takes part, we believe that TNF-α will not be a specific marker for UFs, but our results may be a starting point for further studies. It is possible that the consideration of other biochemical parameters, such as 25-hydroxyvitamin D or TGF-β3 serum levels, in addition to serum TNF-α levels, could increase specificity [26,28,211].

Even if the concept was wrong, it should also be considered whether, for example, TNF alpha could be used as a potential therapy efficacy marker, as with our idea about TGF-β3 [26,28]. Since patients with UFs have elevated TNF-α serum levels, it seems logical to use them as indicators of patient response to various treatment methods. In our opinion, some of the research on UFs should focus on checking which growth factors and cytokines are most frequently associated with specific clinical conditions caused by these tumors. In the case of confirming the dependence of specific symptoms with specific growth factors (e.g., like TNF-α with pain [157,158], therapies for individual patients could be selected more effectively. However, the necessary prerequisite is to investigate various effects of selected drugs on the growth factors, which will allow to choose the best therapy (Figure 5). At this point, again, the validity of the concept of co-drugs in the treatment of UFs should be emphasized. If safe drug connections were found, a broader spectrum of symptoms could be better eliminated, e.g., one drug decreases TGF-β3 levels and slows down the ECM formation, whereas another drug decreases TNF-α level and has a beneficial effect on pain and infertility (Figure 5). Further extensive research in this field is necessary.

## 4. Conclusions

TNF-α is an extremely important cytokine associated with the biology of UFs, UF-related symptoms and complaints. Its concentration has been proven to be elevated in women with clinically symptomatic UFs. The presented data suggest the presence of an “inflammation-like” state in women with UFs where TNF-α is a potent inflammation inducer. The origin of numerous symptoms reported by women with UFs can be traced back to the TNF-α dependent pathways.

Nevertheless, our knowledge on this subject remains limited. It seems vital to study the pathophysiological pathways dependent on TNF-α, in particular its associations with progesterone and activin A. Hopefully, the results of that research will be the decisive factor in selecting the appropriate, individually tailored UF treatment methods. It is possible that TNF-α will prove useful as an additional clinical marker for the diagnosis and therapy of UFs. The importance of anti-TNF-α drugs in the treatment of UFs and how drugs with proven anti-UF action affect the TNF-α dependent symptoms should be investigated further.

## Figures and Tables

**Table 1:** Examples of human diseases with dysregulated TNF-α production [30,50,53,54,55,56,57,58,59].

Field	Examples
Rheumatology	Rheumatoid arthritis Psoriatic Arthritis Ankylosing Spondylitis
Dermatology	Plaque psoriasis
Ophtalmology	Uveitis
Psychiatry	Depression
Gastroenterology	Crohn’s Disease Ulcerative Colitis
Urology	Renal cell carcinoma
Gynecology	Ovarian cancer Uterine fibroids
Neurology	Alzheimer’s Disease

## Abbreviations

ADAM17	ADAM metallopeptidase domain 17
AMH	anti-Müllerian hormone
AP-1	activator protein 1
BMP	bone morphogenetic protein
COX-2	cyclooxygenase 2
ECM	extracellular matrix
EGF	endothelial growth factor
ER	estrogen receptor
ERK	extracellular signal–regulated kinase
FADD	Fas-associated protein with death domain
FAK	focal adhesion kinase
FGF	fibroblast growth factor
GnRH	gonadotropin-releasing hormone
IFN	Interferon
IGF-1	insulin-like growth factor 1
IKK	I kappa B kinase
IL	Interleukin
JNK	c-jun N-terminal kinase
LMS	leiomyosarcoma
MAPK	mitogen-activated protein kinases
MEKK	mitogen-activated protein kinase kinase kinase
MMP	matrix metalloproteinase
NF-κB	nuclear factor kappa-light-chain-enhancer of activated B cells
NIK	NF-kappa-B-inducing kinase
NK	natural killer
NSAID	nonsteroidal anti-inflammatory drug
PCOS	polycystic ovary syndrome
PDGF	platelet-derived growth factor
PG	Prostaglandin
PR	progesterone receptor
RIP	receptor interacting protein
ROS	reactive oxygen species
SHBG	sex hormone-binding globulin
SODD	silencer of death domains
SPRM	selective progesterone receptor modulator
STUMP	smooth muscle tumor of uncertain malignant potential
TAK	TGF-β-activated kinase
TGF-β	transforming growth factor β
TIMP	tissue inhibitor of metalloproteinase
TNFR	TNF-α receptor
TNF-α	tumor necrosis factor α
TRADD	tumor necrosis factor receptor type 1-associated death domain
TRAF	tumor necrosis factor receptor-associated factor
UF	uterine fibroid
UPA	ulipristal acetate
VEGF	vascular endothelial growth factor
